# The IclR-Family Regulator BapR Controls Biofilm Formation in *B. cenocepacia* H111

**DOI:** 10.1371/journal.pone.0092920

**Published:** 2014-03-21

**Authors:** Claudio Aguilar, Nadine Schmid, Martina Lardi, Gabriella Pessi, Leo Eberl

**Affiliations:** Department of Microbiology, Institute of Plant Biology, University of Zurich, Zurich, Switzerland; Ghent University, Belgium

## Abstract

In *Burkholderia cenocepacia* H111, the large surface protein BapA plays a crucial role in the formation of highly structured communities, known as biofilms. We have recently demonstrated that quorum sensing (QS) is necessary for the maximal expression of *bapA*. In this study we identify BapR, a protein from the IclR family of transcriptional regulators that, in conjunction with QS, controls biofilm formation by affecting the expression of *bapA*. We present evidence that, in addition to *bapA*, BapR influences the expression of extracellular proteases, swimming motility and has a profound impact in the incidence of persister cells, making this regulator an interesting target for persister cells and biofilm eradication.

## Introduction

*Burkholderia cenocepacia* is a Gram-negative opportunistic pathogen that belongs to the *Burkholderia cepacia* complex (Bcc), a group that currently comprise 17 bacterial species [1]. Bcc strains show a remarkable ability to thrive in different niches that range from environmental to human clinical settings [2]. Despite having a high potential in biotechnological applications, their use has been restricted due to the emergence of Bcc strains as human opportunistic pathogens, particularly in patients affected by cystic fibrosis [3]–[5].

As part of the mechanisms controlling gene expression, *B. cenocepacia* utilizes QS, an ubiquitous mechanism among Gram-negative bacteria that relies in the synthesis, diffusion, detection and response to self-generated signals [6]. *B. cenocepacia* H111 has two QS systems, one based on *N*-acyl-homoserine lactone (AHL) and a second based on *cis*-2-dodecenoic acid (BDSF) [7]–[9]. These two systems regulate a specific and an overlapping set of genes [9], modulating the expression of phenotypes as diverse as protease production, swarming motility, pathogenicity and the formation of biofilms. Recently, the role of QS-regulated factors that had an impact on biofilm development was studied in *B. cenocepacia* H111 [7]. Among a set of 48 genes identified as downregulated in a *cepR* deficient strain, the lectin cluster BclACB and particularly the large surface protein BapA showed a significant contribution to the development of the biofilm [7]. We sought to extend these findings by looking for additional regulatory elements that could participate in the control of the biofilm phenotype. Here, we identify BapR, a transcriptional regulator of the IclR family that is able to modulate the expression of *bapA* and thus control biofilm formation. We show that BapR, in conjunction with the AHL-BDSF QS systems, is necessary for maximal expression of *bapA* and for maximal biofilm formation. Additionally, we provide evidence that BapR plays a role in the expression of other phenotypes like motility, protease production and also in the maintenance of a persister cell subpopulation of *B. cenocepacia* H111.

## Results and Discussion

### A mutation in CCE51534 results in lowered P*_bapA_-lacZ* expression

We have recently established that cell-to-cell communication in *Burkholderia cenocepacia* H111, mediated by AHLs and by BDSF, controls the expression of a specific and an overlapping set of genes [7], [9]. One of the genes identified in these studies as controlled by both systems was *bapA*, which codes for a large surface protein of crucial importance in biofilm formation [7]. The expression of *bapA* was shown to be diminished in both *cepI* (AHL biosynthesis) or *rpfF_Bc_* (BDSF biosynthesis) mutant backgrounds, and was only restored to wild-type levels when the media was supplemented with both AHLs and BDSF in a double *cepI rpfF_Bc_* mutant background. These results highlighted that expression of *bapA* requires multiple signals, suggesting a complex regulatory network. In order to extend these findings, we aimed to further characterize the regulation of *bapA*, looking for additional regulatory elements that may be part of the transcriptional arsenal driving expression of this gene. Here, we performed transposon mutagenesis in a *B. cenocepacia cepI rpfF_Bc_* double mutant background and screened for diminished activity of P*_bapA_-lacZ* fusion in the presence of BDSF (see methods). From about 86000 clones, 19 clones representing 13 different loci had lowered or no P*_bapA_-lacZ* activity (Table S1, Figure S1). Among the mutants analyzed, we found 3 transposon insertions in the gene CCE51534. This gene codes for a protein of the IclR family of transcriptional regulators that we re-named to *bapR* (*bapA* regulator, see below). Members of this family of proteins can act as transcriptional activators or repressors and may bind cofactor molecules in order to act as DNA-binding regulators [10]. The translated nucleotide sequence of *bapR* was used to search against the genomic database of current members of the Bcc. We found that BapR is ubiquitously distributed within the Bcc, showing the highest identity with the homolog present in the ET12 lineage strain J2315 (Table 1). Interestingly, in this strain *bapR* contains a mutation at position 139 (G to T) that results in an early termination codon, suggesting that BapR is not functional in this strain.

**Table 1:** BapR identity within members of the Bcc.

Bcc. strain	protein ID	% identity^a^
*Burkholderia cenocepacia* J2315	n.d.^b^	99
*Burkholderia cenocepacia* BC7	U1ZF62	99
*Burkholderia cenocepacia* PC184	A2VRI9	98
*Burkholderia cenocepacia* MC0-3	ACA90348.1	98
*Burkholderia cenocepacia* HI2424	ABK07951.1	97
*Burkholderia cenocepacia* AU 1054	ABF75630.1	97
*Burkholderia cenocepacia* KC-01	V4ZZ24	98
*Burkholderia dolosa* AUO158	A2WBB1	96
*Burkholderia multivorans* ATCC 17616	BAG43081.1	94
*Burkholderia multivorans* ATCC 17616	ABX15788.1	94
*Burkholderia multivorans* ATCC BAA-247	J5BML4	95
*Burkholderia multivorans* CGD2M	B9CGL9	95
*Burkholderia multivorans* CGD2	B9BXD9	95
*Burkholderia multivorans* CF2	J5BGZ7	95
*Burkholderia ambifaria* MEX-5	B1TDI7	99
*Burkholderia ambifaria* IOP40-10	B1FMA5	95
*Burkholderia ambifaria* MC40-6	ACB63572.1	94
*Burkholderia ambifaria* AMMD	ABI86640.1	93
*Burkholderia vietnamiensis* G4	ABO54125.1	93
*Burkholderia vietnamiensis* AU4i	U2HJ07	97

a Identity to full-length predicted protein. The predicted BapR protein sequence from *B. cenocepacia* H111 was used to search against the Burkholderia Genome Database (www.burkholderia.com) with the TBLASTN algorithm [20].

b n.d., not determined. In *B. cenocepacia* J2315 there is an ochre, nonsense mutation after codon 46.

### *bapR* expression is not auto-regulated

**Figure 1:**
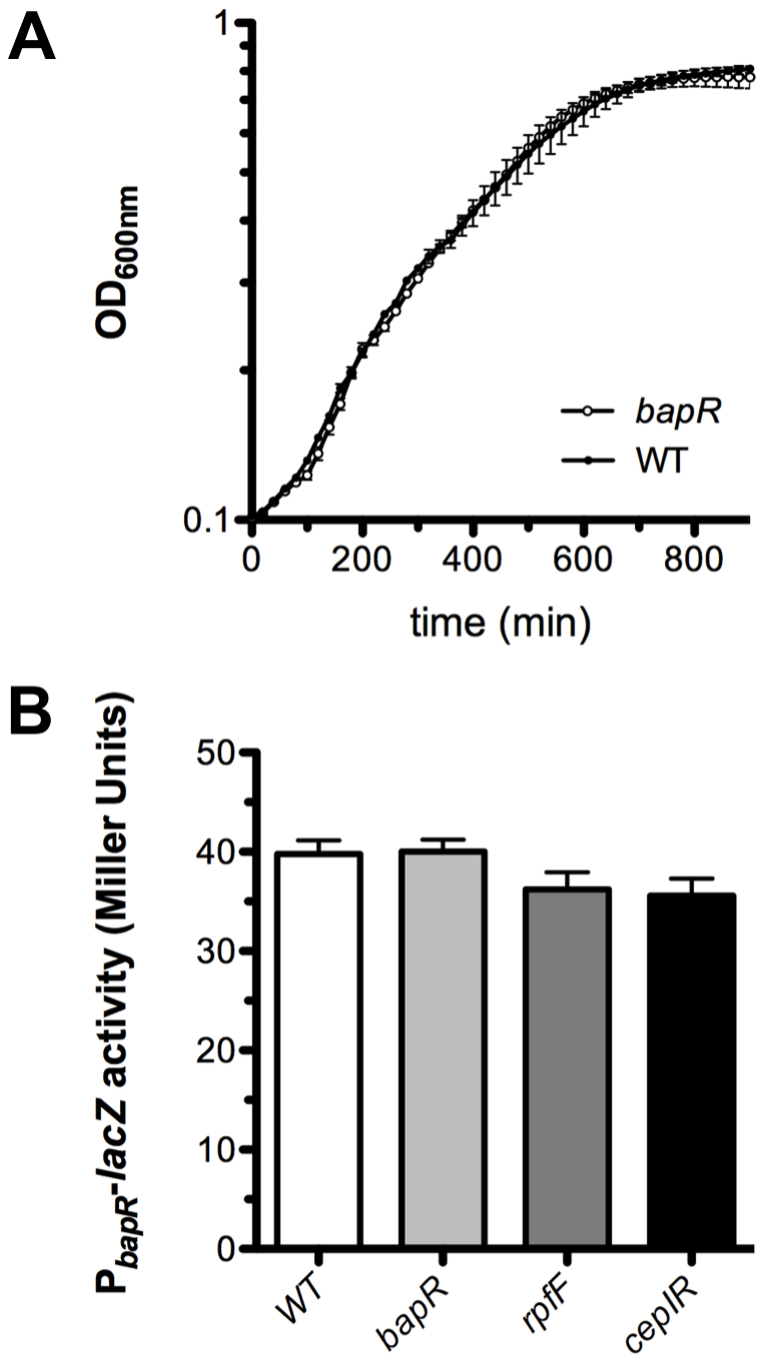
Expression of *bapR* is not auto-regulated. **A**, growth curves of the *bapR* mutant and its parent, WT strain. The strains were inoculated on microtiter plates in LB at 37°C and the OD600nm was monitored every 20 min. No significant differences in growth were detected between the strains (Bars, SEM; n = 3). **B**, the promoter region of *bapR* was cloned in front of a promoter-less *lacZ* reporter gene. The activity of the promoter fusion was measured in different genetic backgrounds, as depicted, using samples obtained from late exponential cultures. No significant difference was detected between the samples (ANOVA, p>0.05). Bars, SEM; n = 3.

We engineered a *bapR* mutant strain and compared its growth to that of the wild-type (WT) strain. As depicted in Figure 1A, no significant differences in growth were detected between these two strains. It has been reported that members of the IclR-family of transcriptional regulators may control their own expression [10] and for this reason we measured the expression of *bapR* using a promoter fusion with *lacZ* as reporter gene in various genetic backgrounds including the *bapR* mutant background. As depicted in Figure 1B, the activity of the *bapR* promoter fusion was comparable in both the WT and the *bapR* mutant background, suggesting that *bapR* is not auto-regulated. Moreover, the activity of the *bapR* promoter was not significantly altered in the QS-deficient mutants *cepIR* or *rpfF_Bc_* (Figure 1B), suggesting that the expression of this gene is not regulated by QS.

### BapR influences the expression of *bapA*

**Figure 2:**
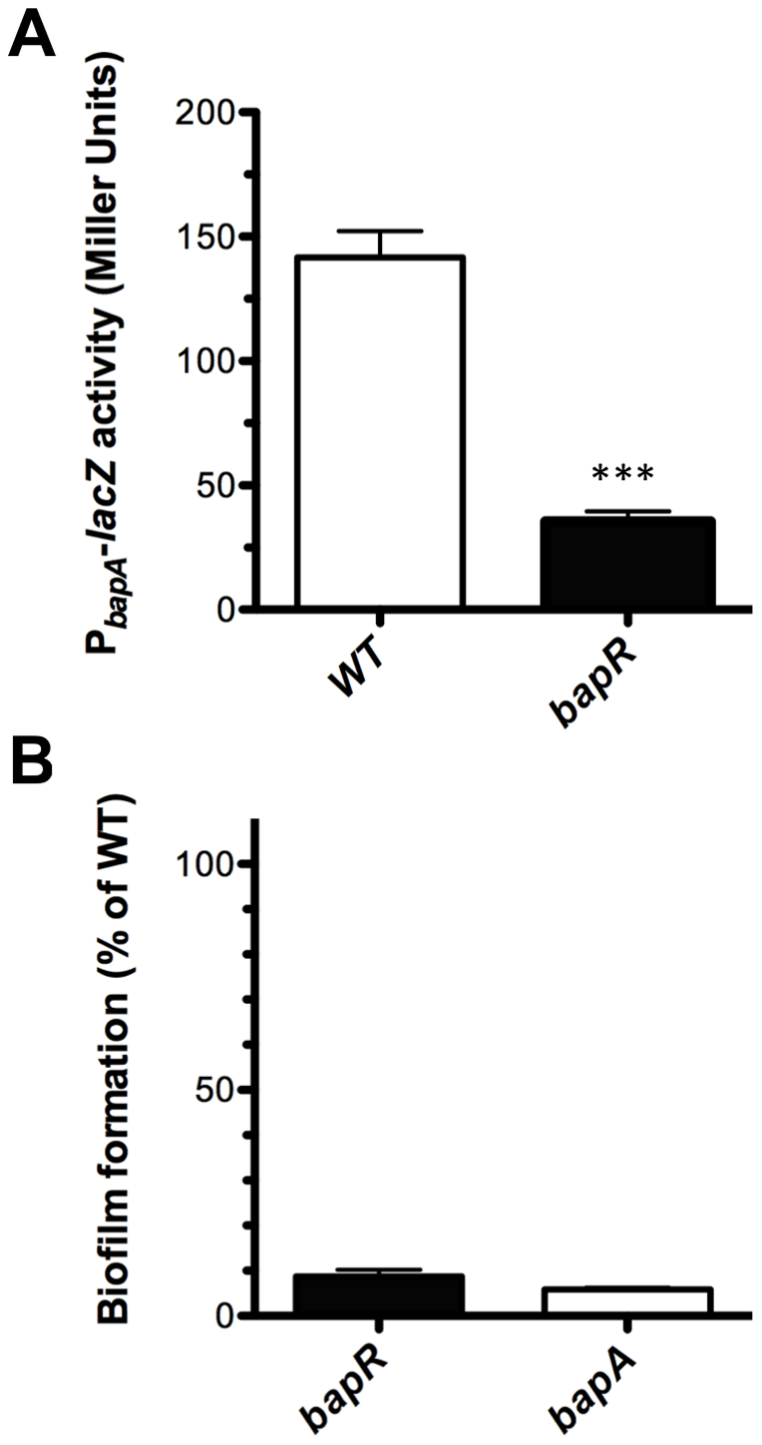
A *bapR* mutation affects *bapA* expression and biofilm formation. **A,** the activity of a P*_bapA_*-*lacZ* promoter fusion was measured in both the WT and the *bapR* genetic backgrounds from samples taken from late exponential cultures. **B**, biofilm formation in 96-well plates was measured after 20 h, staining the biomass adhered to the wells with crystal violet. Error bars, SEM. Asterisks denote significant differences in *bapA* expression between WT and the *bapR* mutant strain (t-test, *** p<0.0001, n≥3).

**Figure 3:**
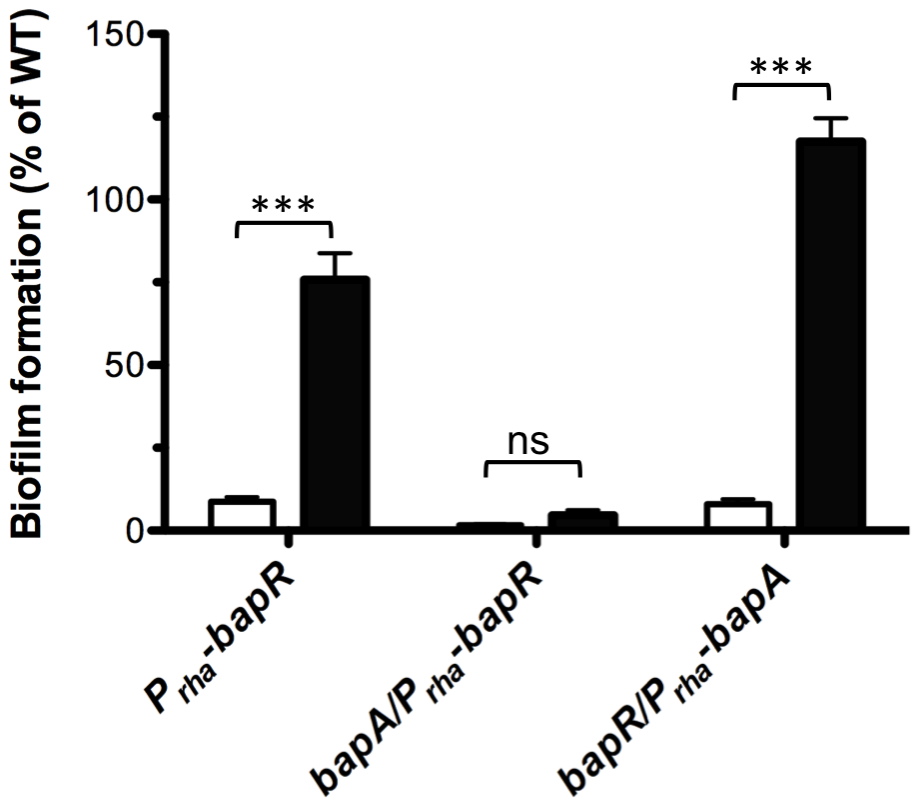
BapR controls biofilm formation via BapA. The defect in biofilm formation of a *bapR* mutant was reverted by expressing *bapR* from a rhamnose-inducible promoter. No biofilm was detected when *bapR* expression was induced in a *bapA* genetic background. Expression of *bapA* is necessary and sufficient to restore the biofilm defect of a *bapR* mutant. White bars are biofilms grown in the absence of rhamnose. Black bars are biofilms grown in the presence of 0.2% rhamnose. Error bars, SEM. Asterisks denote significant differences in biofilm formation when rhamnose was used to induce the expression of *bapR* or *bapA* (2-way ANOVA, *** p<0.001, n≥3).

In order to study and characterize the function of BapR we measured the activity of a P*_bapA_-lacZ* promoter fusion in a *bapR* mutant background. In agreement with the results obtained from the transposon library screening, the activity of the promoter fusion P*_bapA_-lacZ* was diminished in the *bapR* mutant background (Figure 2A). We next tested biofilm formation and found that the *bapR* mutant produced less biofilms compared to the WT (Figure 2B). Based on these results we hypothesized that BapR is necessary for expression of *bapA.* To confirm this hypothesis, we created conditional mutants of *bapR* and *bapA* in which the expression of these genes were induced only upon addition of rhamnose to the media (see methods). We first tested biofilm formation in presence or absence of rhamnose. As depicted in Figure 3, biofilm formation was recovered after expressing *bapR* by the addition of rhamnose. Importantly, no biofilm formation was observed when *bapR* expression was induced in a *bapA* mutant background. We next tested whether the biofilm phenotype persisted when expressing *bapA* in a *bapR* mutant background. In this case, we found that biofilm formation was near WT levels, suggesting that the regulatory action of BapR over *bapA* can be uncoupled and that expression of *bapA* was necessary and sufficient for the formation of biofilms under the conditions tested.

### Maximal expression of *bapA* and biofilm formation requires BapR and QS

**Figure 4:**
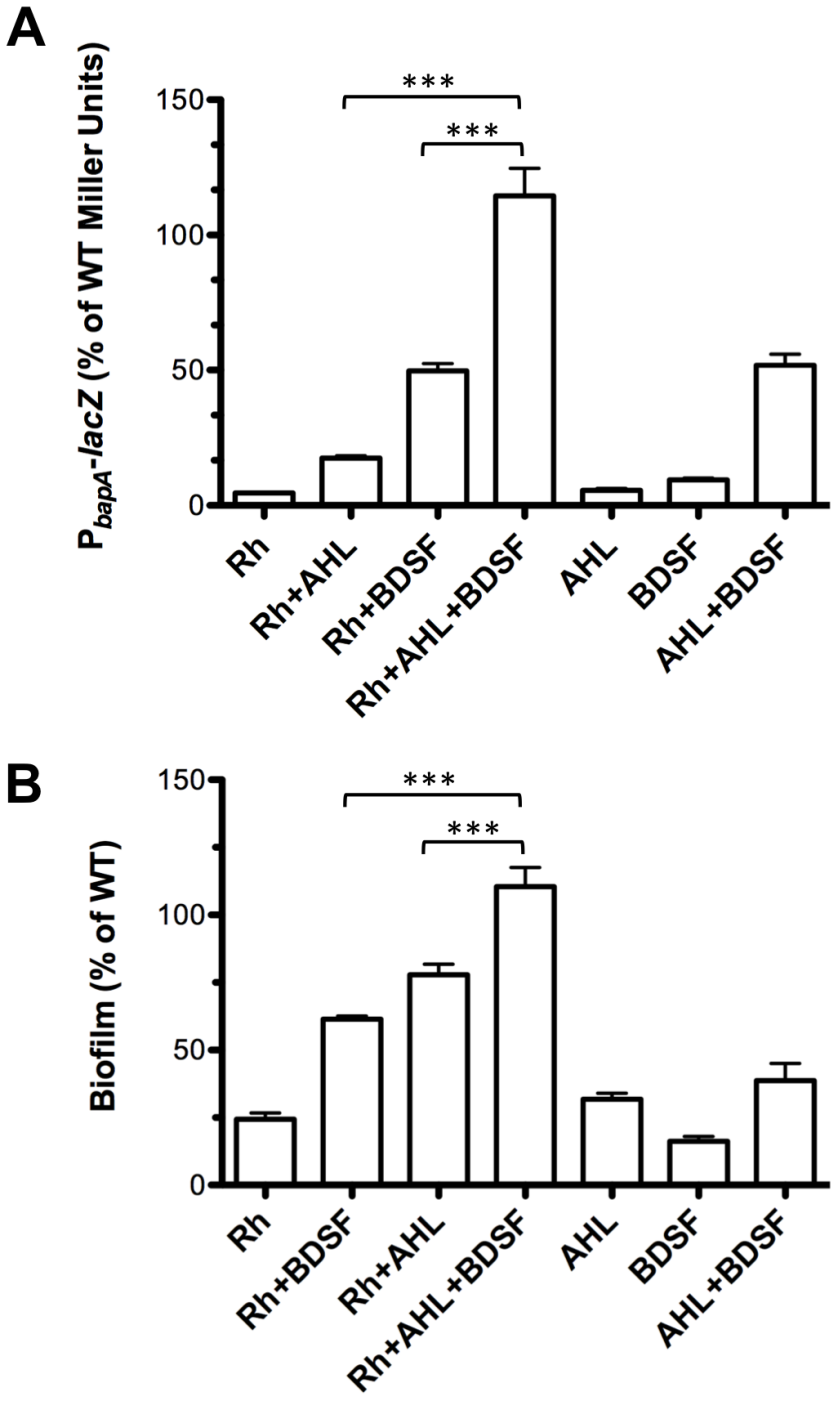
BapR and QS are required for maximal expression of *bapA* and for maximal biofilm formation. **A**, a mutant *cepI rpfF_Bc_*, P*_rha_-bapR* and harboring a P*_bapA_-lacZ* promoter fusion was grown in the presence of rhamnose, C8-AHL, BDSF or the combination of these molecules. Expression of *bapA* was determined measuring the activity of the reporter gene. **B**, biofilm formation was quantified in a mutant *cepI rpfF_Bc_*, P*_rha_-bapR* after the addition of rhamnose, C8-AHL, BDSF or the combination of these molecules. Error bars, SEM. Asterisks denote significant differences in *bapA* expression or in biofilm formation between the samples after supplementing with rhamnose, C8-AHL, BDSF or the combination of these molecules (1-way ANOVA, *** p<0.0001, n≥3).

In a previous study we determined that maximal expression of *bapA* (measured as P*_bapA_-lacZ* activity) was detected only when both AHL and BDSF were present in the medium [9]. We explored whether the regulatory action of BapR over *bapA* required the presence of QS. To do this, we expressed *bapR* from the rhamnose-inducible promoter in a *cepI rpfF_Bc_* double mutant background harbouring a P*_bapA_-lacZ* promoter fusion. As depicted in Figure 4A, maximum activity of the reporter (close to WT levels) was only obtained when *bapR* was induced and both AHL and BDSF were present in the media. We next tested biofilm formation in these conditions. In agreement with the pattern of expression of *bapA*, maximal biofilm formation was obtained only after induction of *bapR* and addition of C8-AHL and BDSF to the media (Figure 4B).

### BapR affects motility, extracellular protease activity and persisters abundance

**Figure 5:**
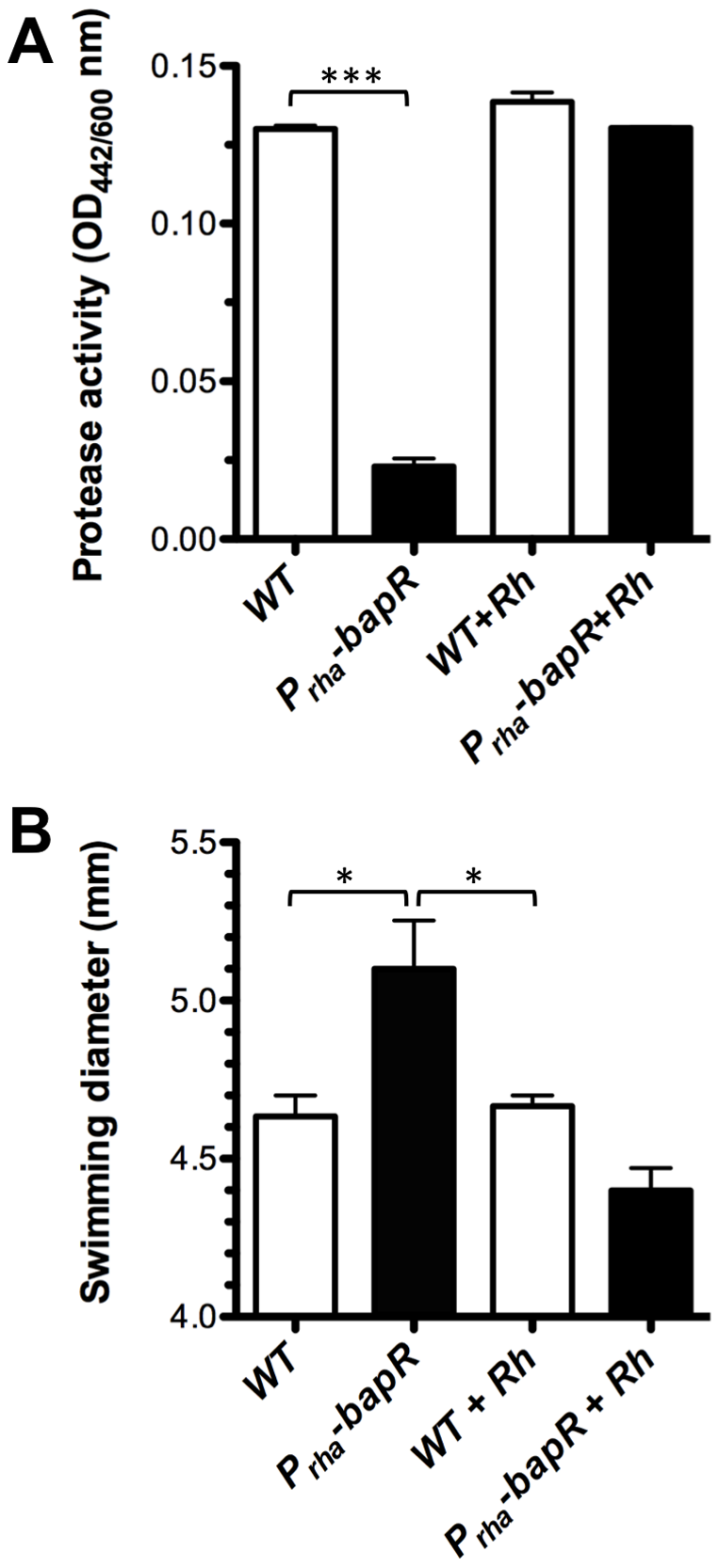
BapR acts positively on extracellular protease activity and negatively on swimming motility. **A**, extracellular protease was measured in the supernatant of WT or *bapR* mutant strains grown in NYG. **B**, swimming motility was measured after incubation of WT or *bapR* mutant strains in LB media fortified with 0.3% agar. Error bars, SEM. Asterisks denote significant differences in protease production or swimming motility between WT and P*_rha_*-*bapR* mutant strain (1-way ANOVA, * p<0.01, *** p<0.0001, n≥3).

**Figure 6:**
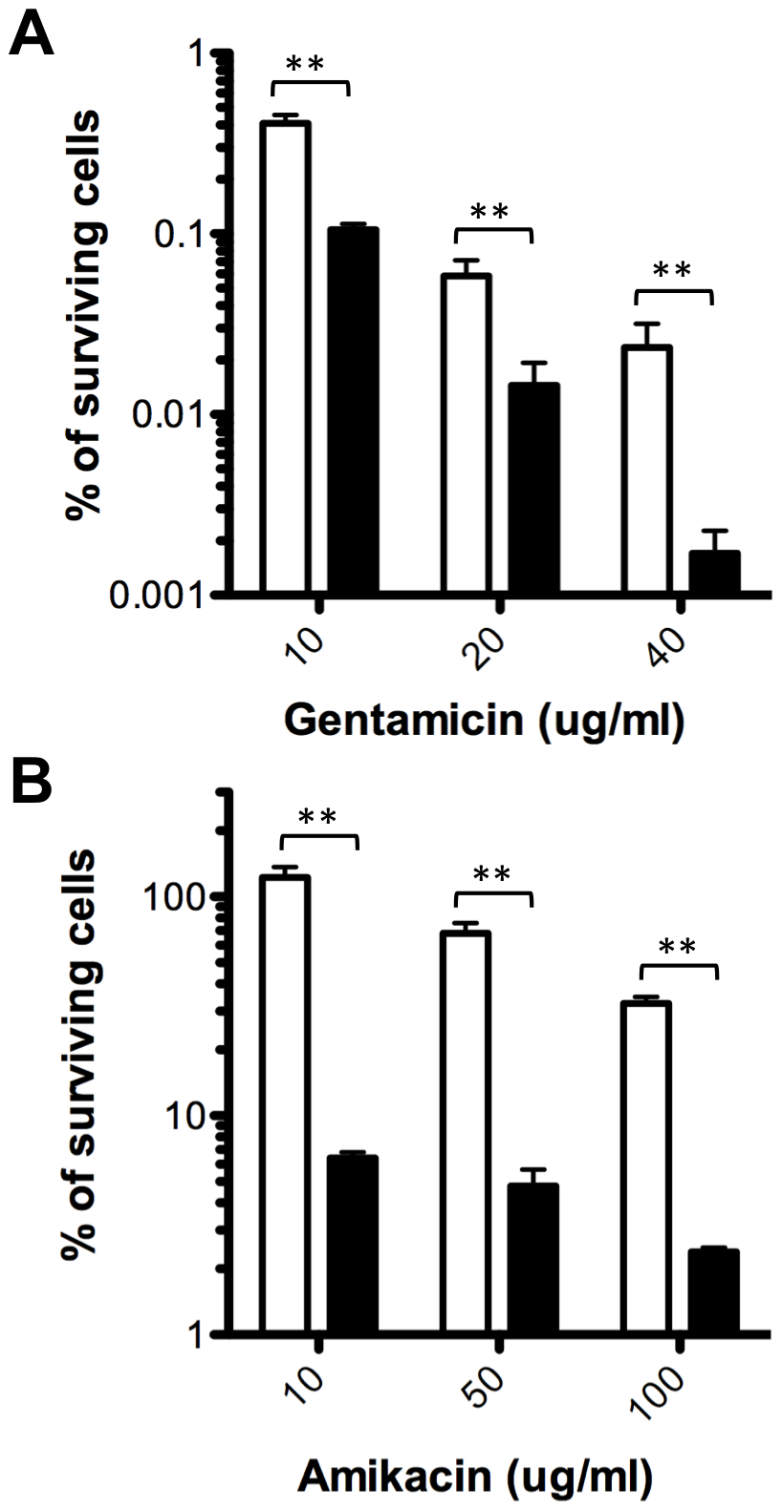
A mutation in *bapR* decreases the number of persister cells. **A**, Percentage of surviving cells after treatment with increasing concentration of gentamicin after 20 h. **B**, Percentage of surviving cells after treatment with increasing concentration of amikacin after 20 h. White bars, WT; black bars, *bapR* mutant. Error bars, SEM. Asterisk denote significant differences in persister cell abundance between WT and *bapR* mutant after treatment with aminoglycoside antibiotics (2-way ANOVA, ** p<0.005, n = 3).

Throughout this study we have provided evidence showing that BapR is an important regulatory element controlling biofilm formation. Since *B. cenocepacia* is an important opportunistic human pathogen, we were interested in exploring the possibility that BapR could also be influencing other aspects of the physiology of this bacterium. For this reason, we performed a transcriptome analysis using RNA-Seq (see Supplementary Methods and Figure S2) which allowed us to compare the global expression of a *bapR* mutant to its respective WT parent strain. The results of this analysis further confirmed that *bapA*, together with all the members of the operon (CCE53117-120) were under the control of BapR (Table S2). Among the top-regulated genes, we found 253 genes with altered expression (116 down-regulated and 119 up-regulated, p<0.1, see Table S2). These genes were classified into different categories and the analysis of these categories suggest that BapR is mainly involved in the control of functions related to amino-acid transport and metabolism (19 genes), cell motility (28 genes), cell wall/membrane/envelope biogenesis (14 genes) and other, non-classified functions (96 genes). Genes showing a diminished expression in a *bapR* mutant background included the *bap* operon (CCE53117-120), the *bclACB* lectins (CCE46720-22), the extracellular protease *zmpB* (CCE52940) and the nematocidal protein *aidA* (CCE52108), which have all been previously described as QS-regulated (Table S2). To further validate the results obtained from this global analysis we chose two conditions and tested for changes in their respective phenotypes. Since ZmpB was found to be regulated by BapR (fold change in expression of -4 between *bapR* mutant and WT, see Table S2) we measured the extracellular protease activity of the *bapR* mutant strain and found that there is in fact a significant decrease (Figure 5A). We also detected that the expression of at least 28 genes involved in cell motility was up-regulated in the *bapR* mutant background. In agreement with these results, the *bapR* mutant displayed a significant increase in motility when tested in a swimming assay (Figure 5B). Interestingly, the expression of a gene coding for an isocitrate lyase (*aceA*, CCE52795, BCAL2118) was down-regulated in the *bapR* mutant background. We validated this result by quantitative PCR, and we could measure a fold-change of −3.1±0.3 in the *bapR* mutant background compared to WT (not shown). A recent study suggested a link between *aceA* and the maintenance of the persister subpopulation of cells in *Bcc* strains [11]. In the clinical setting, chronic, biofilm-related infections are typically refractory to the treatment with antibiotics and it is believed that persister cells are responsible for this recalcitrance [12]. In this context, AceA would be of importance for persister cell maintenance since it helps cells to reduce the production of reactive oxygen species (ROS) by utilizing the glyoxylate shunt instead of the TCA cycle [11]. Motivated by this finding, we next tested whether BapR influenced the abundance of persisters cells in cultures of *B. cenocepacia* H111. As aminoglycoside antibiotics are implicated in the formation of ROS in the cell, we investigated the effect of aminoglycoside antibiotics (gentamicin and amikacin) on the abundance of persister cells in a *bapR* mutant compared to its parent strain. As shown in Figure 6, a mutation in *bapR* has in fact a strong impact on the abundance of persisters upon treatment with aminoglycoside antibiotics, showing a dose-dependent effect that caused a reduction of the persister subpopulation of over 10-fold. These results strongly suggest that in *B. cenocepacia* H111, *aceA* is at least partially responsible for the maintenance of a persister cell sub-population. While we cannot exclude that other genes, in addition to *aceA,* may contribute to the abundance of persister cells, these findings suggest that BapR may represent an interesting drug target for biofilm and persister eradication in the clinic.

In summary, we present evidence that BapR, a novel IclR-family regulator, controls the expression of *bapA* and as a consequence biofilm formation. By means of RNA-seq analysis we show that, in addition to *bapA*, BapR controls a set of approximately 253 genes, including those necessary for swimming, protease production and *aceA*, which plays a role in the maintenance of a persister-cell subpopulation. At the moment it is not known whether BapR controls the expression of target genes directly or *via* downstream regulators. Some of the BapR-controlled phenotypes could be explained by the down-regulation of the QS systems by BapR, like.g. in the case of the BclACB lectins or BapA. However, based on the RNA-seq data, we did not obtained evidence for such a control. We therefore favor the idea that BapR and the QS systems synergistically control biofilm formation and *bapA* expression. Since BapA is a remarkably large protein (over 2700 amino-acid residues), the regulation of *bapA* expression is probably under tight control in the cell. For example, the *bapA* promoter has a long non-translated region of approximately 600 bp which might contain regulatory elements that contribute to *bapA* expression in addition to BapR (Figure S3). The precise regulatory mechanism of BapR and its synergy with the QS system is the subject of ongoing research.

## Materials and Methods

### Bacterial strains, plasmids and growth conditions

Strains and plasmids used in this study are listed in Table S3. Bacterial strains were grown aerobically at 37°C in LB broth, Lennox (BD, Sparks, MD, USA) or AB minimal medium [13] supplemented with 10 mM sodium citrate. Antibiotics were added as required at final concentrations of 100 μg/ml ampicillin, 50 μg/ml kanamycin, 10 μg/ml gentamicin, 100 μg/ml trimethoprim, 80 μg/ml chloramphenicol. Growth was spectrophotometrically monitored by measurement of optical density (OD) at 600 nm.

### DNA manipulations, conjugative plasmid transfer and nucleotide sequencing

Routine DNA molecular techniques were performed using standard methods [14]. Plasmid DNA was isolated with a miniprep kit (Qiagen, Hilden, Germany), chromosomal DNA of *B. cenocepacia* strains was isolated by the sarkosyl-pronase method [15]. Triparental matings from *E. coli* to *B. cenocepacia* were performed with helper strains *E. coli* (pRK600) or *E.coli* (pRK2013) as previously described [16]. Sequencing reactions were performed with the ABI 3730 DNA analyzer using the ABI BigDye Terminator Cycle Sequencing kit (Applied Biosystems, Foster City, USA).

### Transposon mutagenesis and screening

A transposon mutagenesis was performed as previously described [17], using a *cepI rpfF_Bc_* double mutant of *B. cenocepacia* as genetic background and the transposon delivery vector pUT/mini-Tn*5* Km [18]. Approximately 40000 independent transposon insertion mutants were obtained. Aliquots of the library were saved and stored at -80C. To perform the screening, the vector pP*_bapA_*-*lacZ* [7] was introduced into the transposon library by conjugation using *E.coli* S17-1 (pP*_bapA_-lacZ*) as parent strain, selecting for trimethoprim resistant colonies in plates supplemented with X-gal (100 μg/ml) in the presence of BSDF. We supplemented only with BDSF in order to reduce complexity of the system and also since we have determined that addition of this molecule is sufficient to recover approximately 50% of the expression of the promoter fusion in double *cepI rpfF_Bc_* background [9]. Approximately 86000 clones were screened for diminished expression of the P*_bapA_*-*lacZ* promoter fusion. A total of 19 insertions in 13 different loci were found and selected for further analysis. To identify the loci interrupted by the transposon, arbitrary PCR was performed as previously described [17].

### Construction of an insertional mutant in *bapR*

To generate an insertional mutant in *bapR*, a 288 bp internal fragment of CCE51534 was amplified by PCR using oligonucleotides *bapR_*F and *bapR_*R (Table S4) and cloned in pGEMT-easy (Promega). The fragment was then sub-cloned into the suicide vector pEX18Gm as *Bam*HI/*Hind*III fragment, generating pNS-bapR. The plasmid was transferred to *B. cenocepacia* by triparental mating as described, selecting for gentamicin-resistant colonies. The integrity of the insertion was verified by PCR using oligonucleotides bapR-check and pEXcheck_F (Table S4).

### β-galactosidase activity determination

β-galactosidase activity obtained by the product of the *lacZ* reporter gene was quantified as described elsewhere [7]. The oligonucleotides used to create the different promoter fusions are listed in Table S4. Miller Units were obtained with the following formula, which includes normalization by cell growth: MU = (1000*OD_420_/OD_540_)/(time[min]*V[ml]*OD_600_).

### Construction of a rhamnose-inducible *bapR* strain

Using the vector pSC200 [19], *bapR* was engineered for induction of expression upon addition of rhamnose to the media. The vector pSC200 was first digested with *Nde*I (New England Biolabs) and then blunt-ended with Klenow enzyme (Promega). The first 507 bp of *bapR* were amplified by PCR using oligonucleotides CA202 and CA203 with Pfu polymerase (Promega) and then cloned into the blunt-ended vector pSC200. The resulting plasmid, in which a rhamnose-inducible promoter controlled the expression of *bapR*, was transferred to the *B. cenocepacia* H111 by triparental mating [17] and the exconjugants were selected on PIA plates supplemented with trimethoprim. To induce the expression of the promoter, the media was supplemented with 0.2% rhamnose.

### Biofilm quantification

Biofilm formation by *B. cenocepacia* H111 was quantified in a microtiter dish assay as described by Huber *et al*. 2001 with some modifications. Briefly, overnight cultures were normalized to an OD_600_ = 0.05 in AB media supplemented with 10 mM citrate and then used to inoculate a 96-well microtiter dish, incubating statically at 30°C for 20 h. Biofilms were stained by adding 100 μl of a 1% crystal violet solution and incubating for 30 min at room temperature. After the incubation period, the microtiter dishes were inverted to remove the contents of the wells and then they were washed gently and thoroughly using distilled water. The plates were allowed to dry at room temperature. The crystal violet adhered to the wells was resuspended in 120 μl of DMSO and the color was quantified at 550 nm in a Synergy HT microplate reader (Biotek, Luzern, Switzerland).

### Extracellular proteolytic activity determination

Proteolytic activity was quantified as described previously [9]. Briefly, bacteria were grown at 37°C with vigorous shaking to late exponential growth phase in NYG medium (0.5% peptone, 0.3% yeast extract, 2% glycerol) and the OD_600_ was recorded. To start the reaction, 100 μl of a solution of azocasein (5 mg/ml, in 50 mM Tris-Cl, pH 8) was incubated with 100 μl of cell-free supernatant for 60 min at 37°C. After this, 400 μl of 10% TCA were added, followed by centrifugation. The supernatant was mixed with 750 μl of 525 mM NaOH and the absorbance at 442 nm was recorded. Protease activity was expressed as the ratio OD_442_/OD_600_.

### Quantification of persister cells

To quantify the persister cell sub-population, overnight cultures were normalized to an OD_600_ of 0.05 and grown in LB broth with constant shaking until late stationary phase. Planktonic cells were harvested, washed twice with saline solution (0.9% NaCl) and normalized to an OD_600_ of 1 in saline solution. Cells were incubated without shaking at 37°C in the presence or in the absence of antibiotics for 20 h. After the antibiotic treatment, cells were washed twice with saline solution, serial diluted and seeded on LB plates fortified with 1.5% agar for quantification.

### Statistical analysis of data

Histograms, curves and statistical analyses were performed with Prism, V.5a (www.graphpad.com). ANOVA analyses were conducted using Bonferroni post-test with 95% confidence intervals.

### RNA-seq analysis

A full description of the RNA-seq, sequencing data analysis and quantitative PCR methodologies used in this study is presented in Supplementary methods. The RNA-Seq raw data files are accessible through the GEO Series accession number GSE52769.

## Supporting Information

Figure S1 Identification of a mini-Tn*5* transposon insertion in the IclR-type regulator *bapR*. **A**, from the genetic screening, three clones showed a diminished expression of the reporter P*_bapA_-lacZ* at exponential, early and late stationary phases of growth. **B**, Using arbitrary PCR, all three mini-Tn*5* transposon insertion were mapped to a genetic locus coding for an IclR-type transcriptional regulator that we re-named *bapR*.
(TIF)

Figure S2 Differential gene expression in the *bapR* mutant compared to WT. MA plot showing the log2 fold change in expression observed in a *bapR* versus *B. cenocepacia* H111. The top regulated genes are shown in color: genes with increased expression in the *bapR* mutant are indicated in red, whereas genes whose expression was down-regulated are shown in green. Highlighted are *bapA,* the type 1 secretion genes necessary for BapA export (BCAM2142-40) and the protease *zmpB*.
(TIF)

Figure S3 Analysis of the *bapA* promoter region. **A**, the region upstream of *bapA* was systematically analyzed for promoter activity using the *lacZ* reporter gene. Black arrows represent the location and names of the primers used to generate the different promoter fusions, drawn to scale. **B**, β-galactosidase activity of each of the promoter fusions generated using the fragments depicted in **A**, named after the pair of oligonucleotides used in each case. White bars show the activity of the fusion in the WT background. Black bars show the activity of the fusion in the *bapR* mutant background. Error bars, SEM, n = 3.
(TIF)

Methods S1 (DOCX)

Table S1 Genes identified by transposon mutagenesis in *B. cenocepacia* H111 displaying a diminished activity of the P*_bapA_-lacZ* reporter.
(DOCX)

Table S2 Classification of 235 *B. cenocepacia* H111 genes that showed differential expression in a *bapR* mutant strain compared to the wild-type.
(DOCX)

Table S3 Bacterial strains and plasmids used in this study.
(DOCX)

Table S4 Oligonucleotides used in this study.
(DOCX)
