# Supplementary figures and images for: The IclR-Family Regulator BapR Controls Biofilm Formation in B. cenocepacia H111
Source: PLoS One. 2014 Mar 21;9(3):e92920. doi: 10.1371/journal.pone.0092920 (PMC3962473; doi:10.1371/journal.pone.0092920)

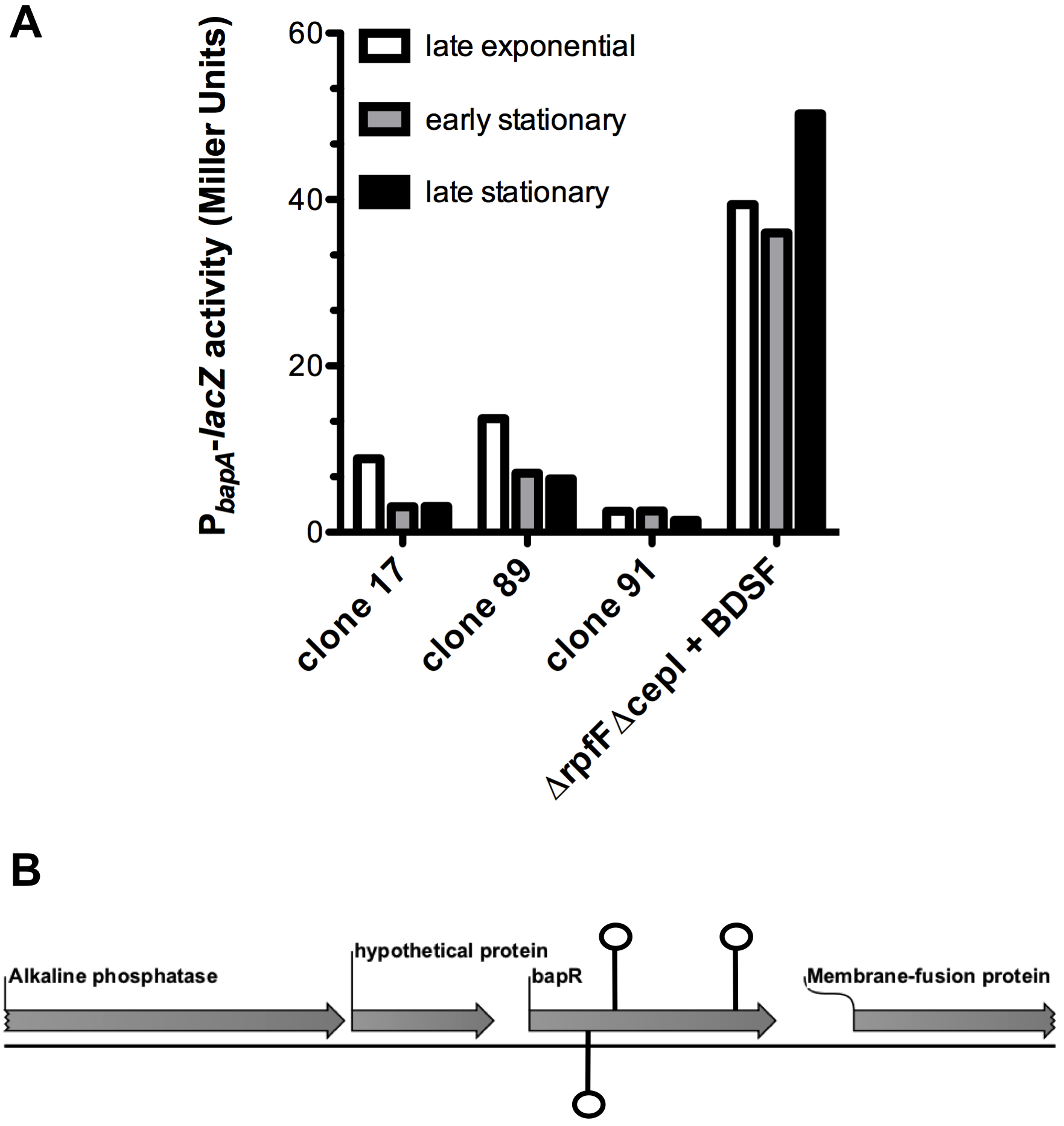

Supplement: Figure S1 — Identification of a mini-Tn5 transposon insertion in the IclR-type regulator bapR. A, from the genetic screening, three clones showed a diminished expression of the reporter PbapA-lacZ at exponential, early and late stationary phases of growth. B, Using arbitrary PCR, all three mini-Tn5 transposon insertion were mapped to a genetic locus coding for an IclR-type transcriptional regulator that we re-named bapR. (TIF) [file pone.0092920.s001.tif]

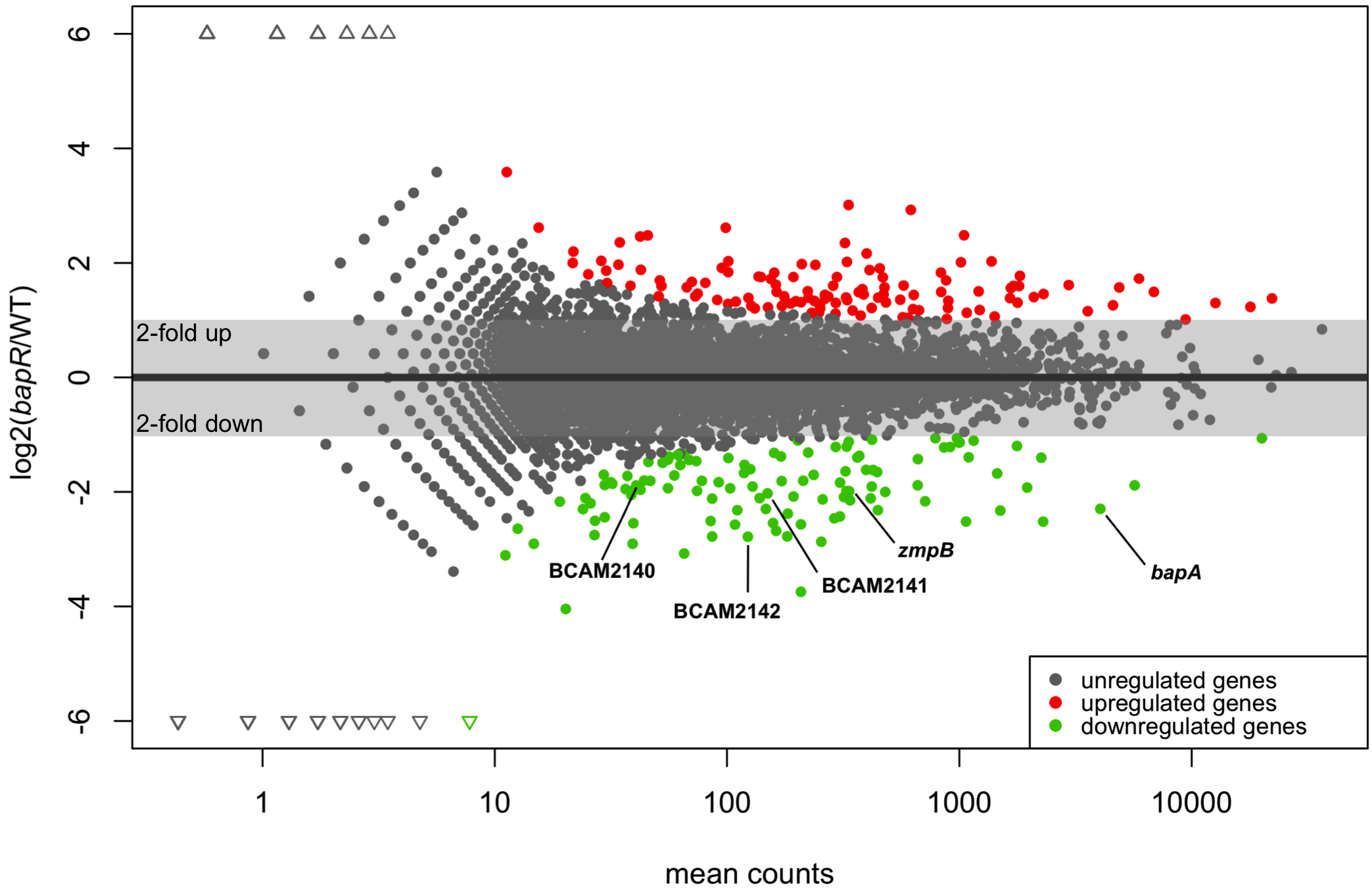

Supplement: Figure S2 — Differential gene expression in the bapR mutant compared to WT. MA plot showing the log2 fold change in expression observed in a bapR versus B. cenocepacia H111. The top regulated genes are shown in color: genes with increased expression in the bapR mutant are indicated in red, whereas genes whose expression was down-regulated are shown in green. Highlighted are bapA, the type 1 secretion genes necessary for BapA export (BCAM2142-40) and the protease zmpB. (TIF) [file pone.0092920.s002.tif]

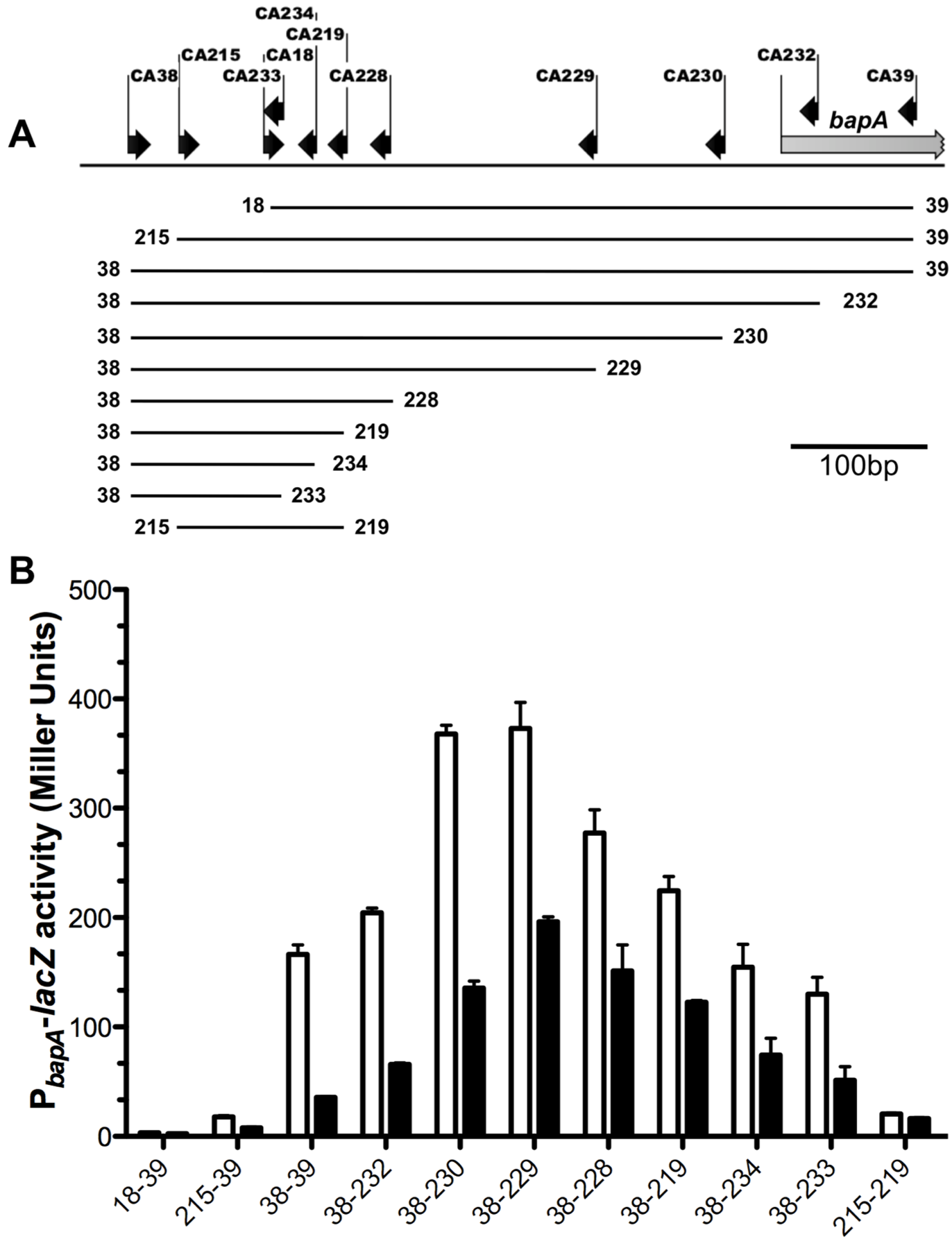

Supplement: Figure S3 — Analysis of the bapA promoter region. A, the region upstream of bapA was systematically analyzed for promoter activity using the lacZ reporter gene. Black arrows represent the location and names of the primers used to generate the different promoter fusions, drawn to scale. B, β-galactosidase activity of each of the promoter fusions generated using the fragments depicted in A, named after the pair of oligonucleotides used in each case. White bars show the activity of the fusion in the WT background. Black bars show the activity of the fusion in the bapR mutant background. Error bars, SEM, n = 3. (TIF) [file pone.0092920.s003.tif]
