# Supplementary material for: The IclR-Family Regulator BapR Controls Biofilm Formation in B. cenocepacia H111
Source: PLoS One. 2014 Mar 21;9(3):e92920. doi: 10.1371/journal.pone.0092920 (PMC3962473; doi:10.1371/journal.pone.0092920)
Supplement: Methods S1 — (DOCX) [file pone.0092920.s004.docx]

**The IclR-Family Regulator BapR Controls Biofilm Formation in *B. cenocepacia* H111.**

Claudio Aguilar, Nadine Schmid, Martina Lardi, Gabriella Pessi and Leo Eberl

**Supplementary Methods**

**Growth conditions, RNA extraction and DNAse treatment**

Cultures of either *B. cenocepacia* H111 or the *bapR* mutant were grown in LB (Lennox) to an OD600 of approx. 1.5 and then used to inoculate 100 ml of LB Lennox medium at an OD600 0.05. Cultures were incubated until late exponential growth phase (OD600 2.0), then 13.5 ml of the culture were rapidly transferred to cold tubes containing 1.5 ml of “stop solution” (10% phenol buffered with 10 mM Tris-HCl, pH 8, in ethanol), centrifuged 5 min at 5000 rpm at 4°C and the pellet frozen in liquid nitrogen. Total RNA was subsequently extracted using a hot acid phenol protocol. Briefly, the pellet was resuspended in 1.5 ml ice-cold buffer A (20 mM sodium acetate pH 5.5, 1 mM EDTA) and added to a mixture of 160 µl 10% SDS, 2 ml buffer A and 3.5 ml acid phenol. The suspension was vigorously mixed for 30 s and incubated for 7 min at 65°C with an additional vortexing step of 1 min in between. The aqueous phase containing the total RNA was separated and re-extracted twice; first with 3 ml of a solution of phenol/chloroform/isoamylalcohol (25:24:1) and then with 2.5 ml chloroform. The total RNA was precipitated at -80°C overnight with 1/10 volume of 3 M sodium acetate pH 5.5 and 2 volumes of 100% ethanol [1] . Quality control for the RNA was performed with RNA Nano Chips (Agilent 2100 Bioanalyzer; RIN >8). To remove 5S rRNA, 50 µg of total RNA in a volume of 100 µl were cleaned using the RNAeasy MiniKit (Qiagen), according to the manufacturer’s protocol, and eluted two times with 30 µl of elution buffer. To remove contaminant DNA in the samples, 30 µg of the RNA were treated with RQ1 RNAse-Free DNAse I (Promega) and SUPERaseIn RNAse Inhibitor (Ambion) for 30 min at 37°C and purified with the RNAeasy MiniKit (Qiagen). The successful depletion of DNA was confirmed by PCR using primers ns38 and ns39 (target: *rhlA*) with 35 cycles.

**Depletion of mRNA, cDNA synthesis and library generation**

The depletion of mRNA was performed by using the MICROB*Express* Kit (Ambion), using 5 µg of RNA, following the instructions provided by the manufacturer. For the synthesis of cDNA and library generation, the Ovation Prokaryotic RNA-Seq System (Nugen) and the Ovation Ultralow Library Systems (Nugen) were used, carefully following the instructions provided by the manufacturer.

**Sequencing and data analysis**

Single-end 100 nucleotide sequence reads were obtained using the Illumina HiSeq 2000 system at the FGCZ (University of Zurich), processed with CASAVA v 1.8.2. The sequencing reads were mapped to the *B. cenocepacia* H111 genome using CLC Genomics v4.9 (CLC bio). The mapped reads were analyzed using the DESeq software as previously described (p-value cut-off ,0.1) [2]. Orthologue mapping and functional classification was performed as described previously [3]. The RNA-Seq raw data files are accessible through the GEO Series accession number GSE52769.

**Quantitative PCR.**

For qPCR analysis, cDNA prepared from biological replicates was used. Each PCR reaction contained 12.5 µl 2x Brilliant III Ultra-Fast SYBR Green QPCR Master Mix, 0.7 µM of individual primers and 3 dilutions of cDNA in a total volume of 25 µl. Relative changes in gene expression were calculated as described elsewhere [4]*.* Expression of the primary sigma factor *rpoD* (BCAM0918) was used as a reference for normalization. Sequences of these primers and of all other oligonucleotides used in this work are available from the authors on request.

**References.**

1. Pessi G, Ahrens CH, Rehrauer H, Lindemann A, Hauser F, et al. (2007) Genome-wide transcript analysis of *Bradyrhizobium japonicum* bacteroids in soybean root nodules. Mol Plant Microbe Interact 20: 1353–1363.

2. Pessi G, Braunwalder R, Grunau A, Omasits U, Ahrens CH, et al. (2013) Response of *Burkholderia cenocepacia* H111 to micro-oxia. PLoS One 8: e72939.

3. Schmid N, Deng Y, Pessi G, Aguilar C, Carlier AL, et al. (2012) The AHL- and BDSF-dependent Quorum Sensing Systems control specific and overlapping sets of genes in *Burkholderia cenocepacia* H111. PLoS One 7: e49966.

4. Pfaffl MW (2001) A new mathematical model for relative quantification in real-time RT-PCR. Nucleic Acids Res 29: e45.
