# Supplementary material for: The IclR-Family Regulator BapR Controls Biofilm Formation in B. cenocepacia H111
Source: PLoS One. 2014 Mar 21;9(3):e92920. doi: 10.1371/journal.pone.0092920 (PMC3962473; doi:10.1371/journal.pone.0092920)
Supplement: Table S1 — Genes identified by transposon mutagenesis in B. cenocepacia H111 displaying a diminished activity of the PbapA-lacZ reporter. (DOCX) [file pone.0092920.s005.docx]

**Table S1**. Genes identified by transposon mutagenesis in *B. cenocepacia* H111 displaying a diminished activity of the P*_bapA_-lacZ* reporter.

| Gene  interrupted in *B.cenocepacia* H111 | Putative function | Orthologue in *B.cenocepacia* J2315 | Number of transposon hits |
| --- | --- | --- | --- |
| CCE50978 | Lipid A core - O-antigen ligase | BCAL0960 | 1 |
| CCE52796 | ATP-dependent RNA helicase | BCAL2117 | 1 |
| CCE49537 | Transcription accessory protein | BCAL1870 | 1 |
| **CCE51534** | **Transcriptional regulator, IclR family (BapR)** | n.f. ^a^ | 3 |
| CCE50110 | zinc-binding protein | n.f. ^a^ | 1 |
| CCE50111 | Mutator mutT protein | BCAL3450 | 1 |
| CCE48324 | Methyl-accepting chemotaxis protein | BCAM0689 | 2 |
| CCE48447 | RpfR | BCAM0580 | 4 |
| CCE48448 | Probable 5-oxoprolinase | BCAM0578 | 1 |
| CCE48451 | Transcriptional regulator, LysR family | BCAM0575 | 1 |
| CCE48474 | Transcriptional regulatory protein ZraR | BCAM0552 | 1 |
| CCE48507 | Chaperonin GroEL | BCAM0525A | 1 |
| CCE48590 | Glutathione S-transferase | BCAM0431 | 1 |

^a^ n.f., not found. No homologue was found in the genome of *B. cenocepacia* J2315.
