# Supplementary material for: The IclR-Family Regulator BapR Controls Biofilm Formation in B. cenocepacia H111
Source: PLoS One. 2014 Mar 21;9(3):e92920. doi: 10.1371/journal.pone.0092920 (PMC3962473; doi:10.1371/journal.pone.0092920)
Supplement: Table S2 — Classification of 235 B. cenocepacia H111 genes that showed differential expression in a bapR mutant strain compared to the wild-type. (DOCX) [file pone.0092920.s006.docx]

**Table S2.** Classification of 235 *B. cenocepacia* H111 genes that showed differential expression in a *bapR* mutant strain compared to the wild-type.

| **Class** | **Locus ID^a^** | **Orthologues J2315** | **Description^b^** | ***WT bapR* vs WT^c^** |
| --- | --- | --- | --- | --- |
| ***Energy production and conversion*** |  |  |  |  |
|  | CCE46507 | BCAL0408 | Phenylacetic acid degradation protein PaaN2 | **-3.8** |
|  | CCE46760 | BCAM0166 | NADH dehydrogenase | **-2.1** |
|  | CCE47517 | BCAM1250 | Acetyl-CoA hydrolase | **2.1** |
|  | CCE48057 | BCAL0206 | Indolepyruvate ferredoxin oxidoreductase | **-3.2** |
|  | CCE49301 | BCAL0064 | Aldehyde dehydrogenase | **-3.2** |
|  | CCE49769 | BCAL2977 | NAD-dependent formate dehydrogenase beta subunit | **-8.6** |
|  | CCE50182 | BCAL2209 | Pyruvate dehydrogenase E1 component | **2.3** |
|  | CCE51010 | BCAL0926 | Aerobic glycerol-3-phosphate dehydrogenase | **3.9** |
|  | CCE51117 | BCAL0206 | Indolepyruvate ferredoxin oxidoreductase, alpha and beta subunits | **-2.2** |
|  | CCE51244 | BCAL3285 | Flavohemoprotein | **-6.9** |
|  | CCE52698 | BCAL2208 | Dihydrolipoamide acetyltransferase | **2.3** |
|  | CCE52699 | BCAL2207 | putative dihydrolipoamide dehydrogenase | **3.0** |
|  | CCE52795 | BCAL2118 | Isocitrate lyase aceA | **-2.8** |
| ***Amino acid transport and metabolism*** |  |  |  |  |
|  | CCE46560 | BCAL0358 | Puromycin-sensitive aminopeptidase | **-2.1** |
|  | CCE46716 | BCAM0190 | Omega-amino acid--pyruvate aminotransferase | **-4.5** |
|  | CCE47472 | BCAM1294 | Dipeptide transport system permease protein DppB | **-2.1** |
|  | CCE47473 | BCAM1293 | ABC-type dipeptide transport system, periplasmic component | **-2.6** |
|  | CCE47474 | BCAM1292 | Dipeptide transport ATP-binding protein DppD | **-2.7** |
|  | CCE48307 | BCAL2019 | putative amino-acid dehydratase | **-3.7** |
|  | CCE49246 | BCAL0110 | Aminotransferase, DegT/DnrJ/EryC1/StrS family | **3.4** |
|  | CCE49351 | BCAL0020 | Leucine-, isoleucine-, valine-, threonine-, and alanine-binding protein | **-3.0** |
|  | CCE49702 | BCAL2902 | AzlC family protein | **3.2** |
|  | CCE49993 | BCAL3183 | Fumarylacetoacetase | **-5.8** |
|  | CCE50287 | BCAM1955 | Gamma-glutamyltranspeptidase | **-4.4** |
|  | CCE51834 | BCAS0055 | Threonine efflux protein | **2.7** |
|  | CCE51908 | BCAS0124 | Putrescine ABC transporter putrescine-binding protein PotF | **-13.4** |
|  | CCE51916 | BCAS0131 | ABC-type proline/glycine betaine transport systems, permease component | **2.3** |
|  | CCE52107 | BCAS0291 | Periplasmic binding protein | **-4.9** |
|  | CCE52939 | BCAM2308 | Bacterial leucyl aminopeptidase | **-5.0** |
|  | CCE52997 | BCAM2251 | Extracellular ligand-binding receptor | **-2.6** |
|  | CCE53001 | BCAM2247 | Branched-chain amino acid transport ATP-binding protein LivF | **-3.6** |
|  | CCE53212 | BCAM2094 | glutamine synthetase family protein | **-3.5** |
| ***Nucleotide transport and metabolism*** |  |  |  |  |
|  | CCE48624 | BCAM0402 | Cytidine/deoxycytidylate deaminase family protein | **4.6** |
| ***Carbohydrate transport and metabolism*** |  |  |  |  |
|  | CCE46473 | BCAM2688 | Mannose-6-phosphate isomerase | **2.4** |
|  | CCE47860 | BCAL1550 | Ribose ABC transport system, ATP-binding protein RbsA | **-3.9** |
|  | CCE47861 | BCAL1549 | Ribose ABC transport system, permease protein RbsC | **-5.7** |
|  | CCE47862 | BCAL1548 | D-ribose-binding protein | **-2.7** |
|  | CCE47863 | BCAL1547 | Fructokinase | **-4.9** |
| ***Coenzyme transport and metabolism*** |  |  |  |  |
|  | CCE46511 | BCAL0404 | Phenylacetate-coenzyme A ligase PaaF | **-2.5** |
|  | CCE46999 | BCAM1711 | Enoyl-CoA hydratase/isomerase | **-2.9** |
|  | CCE49691 | BCAL0979 | Molybdopterin biosynthesis protein MoeA | **-3.5** |
|  | CCE51727 | BCAL0599 | Omega-amino acid--pyruvate aminotransferase | **-3.5** |
| ***Lipid transport and metabolism*** |  |  |  |  |
|  | CCE46509 | BCAL0406 | Phenylacetate degradation enoyl-CoA hydratase PaaA | **-2.6** |
|  | CCE46763 | BCAM0162 | Sterol desaturase | **3.0** |
|  | CCE46998 | BCAM1712 | 3-hydroxybutyryl-CoA dehydrogenase precursor | **-2.7** |
|  | CCE47650 | BCAM2433 | Acyl-CoA dehydrogenase | **-2.5** |
|  | CCE51051 | BCAL0887 | Enoyl-CoA hydratase | **-3.5** |
|  | CCE51052 | BCAL0886 | 3-ketoacyl-CoA thiolase | **-2.6** |
|  | CCE52825 | BCAL0833 | Acetoacetyl-CoA reductase | **-4.0** |
|  | CCE53399 | BCAL0716 | Enoyl-CoA hydratase | **-2.9** |
| ***Transcription*** |  |  |  |  |
|  | CCE46477 | BCAM2684 | GCN5-related N-acetyltransferase | **3.4** |
|  | CCE47267 | BCAM1483 | transcriptional regulator, Crp/Fnr family | **2.6** |
|  | CCE47859 | BCAL1551 | transcriptional regulator/sugar kinase | **-5.9** |
|  | CCE49230 | BCAL0125 | Flagellar transcriptional activator FlhC | **4.5** |
|  | CCE49231 | BCAL0124 | Flagellar transcriptional activator FlhD | **5.6** |
|  | CCE50415 | BCAM1778 | transcriptional regulator, LysR family | **2.7** |
|  | CCE51113 | BCAL0209 | Histone acetyltransferase HPA2 and related acetyltransferases | **2.1** |
| ***Cell wall/membrane/envelope biogenesis*** |  |  |  |  |
|  | CCE50890 | BCAM0861 | Glycosyltransferase | **-5.4** |
|  | CCE50893 | BCAM0858 | Polysaccharide export lipoprotein Wza | **-4.6** |
|  | CCE48728 | BCAM1010 | UTP--glucose-1-phosphate uridylyltransferase | **-4.2** |
|  | CCE50895 | BCAL2946 | UDP-glucose dehydrogenase | **-3.6** |
|  | CCE50892 | BCAM0859 | Tyrosine-protein kinase Wzc | **-3.6** |
|  | CCE48732 | BCAM1008 | Glycosyltransferase | **-2.8** |
|  | CCE53446 | BCAL1674 | RND efflux system, membrane fusion protein CmeA | **-2.6** |
|  | CCE50887 | BCAM0864 | Glycosyltransferase | **-2.5** |
|  | CCE48128 | BCAL0584 | Outer membrane porin protein precursor | **-2.3** |
|  | CCE46634 | BCAL0287 | Outer membrane protein precursor | **-2.2** |
|  | CCE50413 | BCAM1780 | Lipoprotein nlpD | **2.2** |
|  | CCE51621 | BCAL1409 | Acid phosphatase | **3.6** |
|  | CCE52316 | BCAS0460 | Outer membrane porin protein precursor | **3.8** |
|  | CCE47981 | BCAL3508 | CidA-associated membrane protein CidB | **3.9** |
| ***Cell motility*** |  |  |  |  |
|  | CCE48147 | BCAL0564 | Flagellar basal-body rod protein FlgB | **2.3** |
|  | CCE48186 | BCAL0528 | Flagellar biosynthesis protein FliT | **2.3** |
|  | CCE48144 | BCAL0567 | Flagellar hook protein FlgE | **2.4** |
|  | CCE48136 | BCAL0576 | Flagellar hook-associated protein FlgK | **2.5** |
|  | CCE51174 | BCAL0136 | Chemotaxis response-phosphatase CheZ | **2.5** |
|  | CCE48187 | BCAL0527 | Flagellar biosynthesis protein FliS | **2.5** |
|  | CCE48135 | BCAL0577 | Flagellar hook-associated protein FlgL | **2.5** |
|  | CCE48140 | BCAL0571 | Flagellar P-ring protein FlgI | **2.5** |
|  | CCE48141 | BCAL0570 | Flagellar L-ring protein FlgH | **2.5** |
|  | CCE49242 | BCAL0114 | Flagellar biosynthesis protein FliC | **2.6** |
|  | CCE51168 | BCAL0142 | Flagellar biosynthesis protein FlhF | **2.6** |
|  | CCE48146 | BCAL0565 | Flagellar basal-body rod protein FlgC | **2.7** |
|  | CCE48189 | BCAL0525 | Flagellar M-ring protein FliF | **2.7** |
|  | CCE48190 | BCAL0524 | Flagellar motor switch protein FliG | **2.8** |
|  | CCE47983 | BCAL3506 | Flagellar motor switch protein FliM | **3.0** |
|  | CCE48145 | BCAL0566 | Flagellar basal-body rod modification protein FlgD | **3.0** |
|  | CCE51170 | BCAL0140 | Flagellar biosynthesis protein FlhB | **3.1** |
|  | CCE48142 | BCAL0569 | Flagellar basal-body rod protein FlgG | **3.2** |
|  | CCE49228 | BCAL0126 | Flagellar motor rotation protein MotA | **3.3** |
|  | CCE47982 | BCAL3507 | Flagellar biosynthesis protein FliL | **3.4** |
|  | CCE48188 | BCAL0526 | Flagellar hook-basal body complex protein FliE | **3.6** |
|  | CCE48143 | BCAL0568 | Flagellar basal-body rod protein FlgF | **3.7** |
|  | CCE48191 | BCAL0523 | Flagellar assembly protein FliH | **3.7** |
|  | CCE51169 | BCAL0141 | Flagellar biosynthesis protein FlhA | **3.7** |
|  | CCE48909 | BCAM0777 | Flagellar motor rotation protein MotA | **5.6** |
|  | CCE48908 | BCAM0778 | Flagellar motor rotation protein MotB | **8.1** |
|  | CCE51178 | BCAL0132 | Chemotaxis protein methyltransferase CheR | **2.3** |
|  | CCE46492 | BCAL0130 | Positive regulator of CheA protein activity | **2.7** |
| ***Posttranslational modification, protein turnover, chaperones*** |  |  |  |  |
|  | CCE51461 | BCAL1234 | Molecular chaperone (small heat shock protein) | **-7.3** |
|  | CCE52629 | BCAL3146 | Heat shock protein 60 family chaperone GroEL | **-5.7** |
|  | CCE51462 | BCAL1233 | Molecular chaperone (small heat shock protein) | **-4.3** |
|  | CCE52630 | BCAS0638 | Heat shock protein 60 family co-chaperone GroES | **-4.2** |
|  | CCE49951 | BCAL3147 | Heat shock protein 60 family co-chaperone GroES | **-2.2** |
|  | CCE49950 | BCAL3146 | Heat shock protein 60 family chaperone GroEL | **-2.1** |
|  | CCE46962 | BCAM1744 | Extracellular protease precursor | **2.8** |
|  | CCE49245 | BCAL0111 | putative O-linked N-acetylglucosamine transferase | **3.0** |
|  | CCE53056 | BCAM2199 | putative stomatin/prohibitin-family membrane protease | **3.6** |
|  | CCE48911 | BCAM0775 | Glutathione S-transferase | **3.6** |
|  | CCE53057 | BCAM2198 | putative membrane-bound ClpP-class protease | **6.1** |
| ***Inorganic ion transport and metabolism*** |  |  |  |  |
|  | CCE50475 | BCAL2352 | Carbonic anhydrase | **-5.0** |
|  | CCE53425 | BCAL1692 | Ferrichrome transport system permease protein fhuB | **-4.3** |
|  | CCE49583 | BCAL1700 | Ferrichrome-iron receptor | **-3.8** |
|  | CCE48638 | BCAM0389 | Alkaline phosphatase | **2.3** |
|  | CCE46340 | BCAM2496 | 2-aminoethylphosphonate ABC transporter permease protein | **2.4** |
|  | CCE48637 | BCAM0390 | Alkaline phosphatase | **2.9** |
|  | CCE48625 | BCAM0401 | Cyanate hydratase | **3.5** |
|  | CCE52312 | BCAS0457 | Alkylphosphonate utilization operon protein PhnA | **5.5** |
|  | CCE49584 | BCAL1699 | L-ornithine 5-monooxygenase | **-8.4** |
| ***Secondary metabolites biosynthesis, transport and catabolism*** |  |  |  |  |
|  | CCE49994 | BCAL3184 | Homogentisate 1,2-dioxygenase | **-5.9** |
|  | CCE46710 | BCAM0195 | Peptide synthetase | **-5.0** |
|  | CCE53427 | BCAL1690 | SyrP-like protein | **-3.9** |
|  | CCE46715 | BCAM0191 | Enterobactin synthetase component F | **-3.7** |
|  | CCE51107 | BCAL0215 | Phenylacetate-CoA oxygenase, PaaH subunit | **-3.1** |
|  | CCE51115 | BCAL0207 | 4-hydroxyphenylpyruvate dioxygenase | **2.2** |
|  | CCE46422 | BCAM2742 | Aromatic ring-cleaving dioxygenase | **3.4** |
|  | CCE50894 | BCAM0857 | Low molecular weight protein-tyrosine-phosphatase Wzb | **-7.5** |
| ***Signal transduction mechanisms*** |  |  |  |  |
|  | CCE47247 | BCAM1503 | Methyl-accepting chemotaxis protein | **2.0** |
|  | CCE51176 | BCAL0134 | Chemotaxis response regulator protein-glutamate methylesterase CheB | **2.1** |
|  | CCE47328 | BCAM1424 | Methyl-accepting chemotaxis protein | **2.2** |
|  | CCE46472 | BCAM2689 | Methyl-accepting chemotaxis protein I | **2.6** |
|  | CCE47576 | BCAM1195 | Signal transduction histidine kinase CheA | **2.6** |
|  | CCE47266 | BCAM1484 | Response regulator, CheY-like domain and an HTH DNA-binding domain | **2.8** |
|  | CCE53354 | BCAL0762 | Methyl-accepting chemotaxis protein I | **2.8** |
|  | CCE50388 | BCAM1804 | Methyl-accepting chemotaxis protein I | **2.9** |
|  | CCE46493 | BCAL0129 | Signal transduction histidine kinase CheA | **3.0** |
|  | CCE46418 | BCAM2746 | Carbon starvation protein A | **3.4** |
|  | CCE47169 | BCAM1572 | Methyl-accepting chemotaxis protein I | **4.1** |
|  | CCE49582 | BCAL1701 | Pyoverdine synthetase PvdF, N5-hydroxyornithine formyltransferase | **-4.5** |
| ***Others*** |  |  |  |  |
|  | CCE53117 | BCAM2143 | BapA | **-4.9** |
|  | CCE53118 | BCAM2142 | Type I secretion system | **-6.9** |
|  | CCE53119 | BCAM2141 | Type I secretion system | **-4.1** |
|  | CCE53120 | BCAM2140 | Type I secretion system | **-3.7** |
|  | CCE46722 | BCAM0184 | lectin BclB | **-5.9** |
|  | CCE46721 | BCAM0185 | lectin BclC | **-4.3** |
|  | CCE46720 | BCAM0186 | lectin BclA | **-6.9** |
|  | CCE52940 | BCAM2307 | extracellular protease ZmpB | **-4.0** |
|  | CCE52109 | BCAS0293 | AidA | **-3.7** |
|  | CCE52108 | BCAS0292 | AidA | **-4.4** |
|  | CCE46207 |  | outer membrane protein | **2.0** |
|  | CCE46295 | BCAM2536 | Alpha/beta hydrolase fold protein | **3.0** |
|  | CCE46419 | BCAM2745 | hypothetical small protein yjiX | **3.2** |
|  | CCE46421 | BCAM2743 | hypothetical protein I35_0224 | **2.7** |
|  | CCE46491 |  | hypothetical protein I35_0296 | **2.5** |
|  | CCE46708 | BCAM0197 | transcriptional regulator, LysR family | **-5.2** |
|  | CCE46709 | BCAM0196 | hypothetical protein I35_0519 | **-5.4** |
|  | CCE46711 | BCAM0194 | hypothetical protein I35_0521 | **-5.5** |
|  | CCE46712 | BCAM0193 | hypothetical protein I35_0522 | **-6.4** |
|  | CCE46713 | BCAM0192 | hypothetical protein | **-4.3** |
|  | CCE46717 | BCAM0189 | transcriptional regulator, AraC family | **-3.1** |
|  | CCE46778 | BCAM0147 | hypothetical protein I35_0588 | **-3.8** |
|  | CCE46823 | BCAM0078 | NADH dehydrogenase | **-2.5** |
|  | CCE46827 |  | hypothetical protein I35_0637 | **3.0** |
|  | CCE46961 |  | hypothetical protein I35_0773 | **4.1** |
|  | CCE46963 |  | hypothetical protein I35_0775 | **2.8** |
|  | CCE46965 | BCAM1742 | hypothetical protein I35_0777 | **-2.7** |
|  | CCE46973 | BCAM1736 | Glucose dehydrogenase, membrane-bound, gamma subunit | **-2.2** |
|  | CCE47152 |  | hypothetical protein I35_0964 | **4.1** |
|  | CCE47248 | BCAM1502 | hypothetical protein I35_1060 | **2.2** |
|  | CCE47258 | BCAM1491 | hypothetical protein I35_1070 | **2.4** |
|  | CCE47394 |  | hypothetical protein I35_1206 | **-3.7** |
|  | CCE47408 | BCAM1351 | DnaK suppressor protein | **5.1** |
|  | CCE47516 |  | Multidrug resistance protein B | **2.5** |
|  | CCE47525 |  | hypothetical protein I35_1337 | **3.1** |
|  | CCE47571 | BCAM1200 | hypothetical protein I35_1383 | **4.1** |
|  | CCE47572 | BCAM1199 | Chemotaxis regulator | **6.1** |
|  | CCE47573 | BCAM1198 | hypothetical protein I35_1385 | **5.1** |
|  | CCE47574 | BCAM1197 | hypothetical protein I35_1386 | **2.3** |
|  | CCE47575 | BCAM1196 | Methyl-accepting chemotaxis protein | **2.8** |
|  | CCE47661 | BCAM2425 | hypothetical protein I35_1474 | **12.0** |
|  | CCE47730 |  | hypothetical protein I35_1547 | **3.4** |
|  | CCE47883 | BCAL1527 | Flp pilus assembly protein RcpC/CpaB | **2.4** |
|  | CCE47885 | BCAL1525a | putative prepilin peptidase transmembrane protein | **2.6** |
|  | CCE47891 | BCAL1520 | Uncharacterized protein conserved in bacteria | **-2.6** |
|  | CCE47980 | BCAL3509 | Holin-like protein CidA | **2.5** |
|  | CCE48006 | BCAL3485 | hypothetical protein I35_1835 | **3.1** |
|  | CCE48150 | BCAL0561 | Flagellar biosynthesis protein FlgN | **2.2** |
|  | CCE48184 |  | Ribonuclease E | **2.6** |
|  | CCE48320 | BCAM0693 | Histone acetyltransferase HPA2 and related acetyltransferases | **-3.3** |
|  | CCE48385 | BCAM0634 | hypothetical protein I35_2218 | **-3.7** |
|  | CCE48386 | BCAM0633 | Leucyl aminopeptidase | **-2.3** |
|  | CCE48523 |  | hypothetical protein I35_2356 | **-16.5** |
|  | CCE48629 | BCAM0397 | hypothetical protein | **2.5** |
|  | CCE48636 | BCAM0391 | hypothetical protein I35_2469 | **2.6** |
|  | CCE48664 | BCAM0384 | Large exoproteins involved in heme utilization or adhesion | **2.5** |
|  | CCE48907 | BCAM0779 | Methyl-accepting chemotaxis protein | **2.4** |
|  | CCE48910 | BCAM0776 | cAMP-binding protein | **7.6** |
|  | CCE49081 | BCAL2734 | hypothetical protein I35_2929 | **-7.5** |
|  | CCE49088 |  | hypothetical protein I35_2937 | **-4.0** |
|  | CCE49208 |  | hypothetical protein I35_3059 | **2.1** |
|  | CCE49247 |  | hypothetical protein I35_3099 | **2.7** |
|  | CCE49248 |  | hypothetical protein I35_3100 | **3.0** |
|  | CCE49251 |  | 3-demethylubiquinone-9 3-methyltransferase | **3.1** |
|  | CCE49300 | BCAL0065 | hypothetical protein I35_3153 | **-4.3** |
|  | CCE49631 |  | hypothetical protein I35_3490 | **-2.5** |
|  | CCE49632 | BCAL2786 | selenium binding protein, putative | **-2.3** |
|  | CCE49681 | BCAL0989 | hypothetical protein I35_3541 | **2.3** |
|  | CCE49692 | BCAL0978 | putative transmembrane protein | **-3.9** |
|  | CCE49704 | BCAL2904 | hypothetical protein I35_3566 | **2.8** |
|  | CCE50416 | BCAM1777A | hypothetical protein I35_4294 | **4.0** |
|  | CCE50476 | BCAL2353 | Sulfate permease | **-5.7** |
|  | CCE50668 | BCAL2607 | ATPase involved in DNA repair | **-2.3** |
|  | CCE50909 | BCAM0844 | hypothetical protein I35_4795 | **-2.6** |
|  | CCE51106 | BCAL0216 | Phenylacetate-CoA oxygenase, PaaG subunit | **-3.7** |
|  | CCE51108 | BCAL0214 | Phenylacetate-CoA oxygenase, PaaI subunit | **-3.1** |
|  | CCE51109 | BCAL0213 | Phenylacetate-CoA oxygenase, PaaJ subunit | **-3.3** |
|  | CCE51400 | BCAL1296 | VgrG protein | **-2.1** |
|  | CCE51607 | BCAL2308 | hypothetical protein I35_5510 | **2.2** |
|  | CCE51909 |  | hypothetical protein I35_5820 | **-6.7** |
|  | CCE51938 | BCAS0151 | hypothetical protein I35_5849 | **2.6** |
|  | CCE51939 | BCAS0152 | Non-heme chloroperoxidase | **2.7** |
|  | CCE51942 | BCAS0155 | putative cytoplasmic protein | **-3.1** |
|  | CCE52221 | BCAS0398 | GGDEF domain protein | **3.3** |
|  | CCE52315 | BCAS0459 | hypothetical protein I35_6226 | **3.9** |
|  | CCE52628 | BCAS0636 | hypothetical protein I35_6539 | **-3.6** |
|  | CCE52632 | BCAS0640 | hypothetical protein I35_6543 | **-6.3** |
|  | CCE52659 |  | tannase precursor | **-3.3** |
|  | CCE52672 | BCAS0665 | hypothetical protein I35_6583 | **-2.8** |
|  | CCE53121 | BCAM2139 | motif=eukaryotic putative RNA-binding region RNP-1 signature | **-3.5** |
|  | CCE53210 | BCAM2096 | Oxidoreductase | **-2.3** |
|  | CCE53211 | BCAM2095 | transcriptional regulator, MerR family | **-3.3** |
|  | CCE53255 |  | hypothetical protein I35_7186 | **2.7** |
|  | CCE53285 | BCAL0831 | granule-associated protein | **-4.0** |
|  | CCE53353 |  | hypothetical protein I35_7288 | **4.0** |
|  | CCE53428 | BCAL1689 | hypothetical MbtH-like protein, PA2412 homolog | **-5.7** |

^a^ Nomenclature according to GenBank file CAFQ01000001.1

^b^ Description according to the EggNOG classification.

^c^ Fold change (FC) of transcript expression, comparing wild-type strain with *bapR* mutant (DE-Seq analysis, p-value <0.1).
