# Supplementary material for: The IclR-Family Regulator BapR Controls Biofilm Formation in B. cenocepacia H111
Source: PLoS One. 2014 Mar 21;9(3):e92920. doi: 10.1371/journal.pone.0092920 (PMC3962473; doi:10.1371/journal.pone.0092920)
Supplement: Table S3 — Bacterial strains and plasmids used in this study. (DOCX) [file pone.0092920.s007.docx]

**Table S3.** Bacterial strains and plasmids used in this study.

| **Strain or plasmid** | **Description** | **Reference** |
| --- | --- | --- |
| Strains |  |  |
| *E. coli* |  |  |
| DH5α | F^-^ Φ80*lacZ*∆M15 ∆(*lacZYA-argF*) *recA1 endA gyrA96 thi-1 hsdR17 supE44 relAl deoR(U169)* | [1] |
| MM294 | F^-^ *endA1 hsdR17 supE44*(AS*) rfbD1 spoT1 thi-1* | [2] |
| S17-1 | RP4 Mob+ | [3] |
| DB3.1λpir | λpir lysogen of strain DB3.1 | [4] |
| Top10 | Δ*lacX74 ara*Δ*139*Δ*(ara-leu)* | Invitrogen |
| *B. cenocepacia* |  |  |
| H111 | CF isolate from Germany, genomovar III | [5,6] |
| H111- *bapA* | *bapA*::*km* mutant of H111; Km^r^ | [8] |
| H111 Δ*cepI* | Δ*cepI* mutant of H111, markerless | [7] |
| H111 Δ*cepI rpfF_Bc_* | Δ*cepI* and *rpfF_Bc_*::pSHAFT double mutant, Cm^r^ | [7] |
| H111 P*_rha_*-*bapA* | H111 expressing *bapA* from a rhamnose-inducible promoter | [8] |
| H111 *bapR* | *bapR*::pEX18Gm insertional mutant | This study |
| H111 *bapR* P*_rha_*-*bapA* | *bapR*::pEX18Gm insertional mutant expressing *bapA* from a rhamnose-inducible promoter | This study |
| H111 P*_rha_*-*bapR* | H111 expressing *bapR* from a rhamnose-inducible promoter | This study |
| H111 Δ*cepI* *rpfF_Bc_* P*_rha_*-*bapR* | Δ*cepI* and *rpfF_Bc_*::pSHAFT double mutant, expressing *bapR* from a rhamnose-inducible promoter | This study |
| H111 Δ*cepI* *rpfF_Bc_* P*_rha_*-*bapR* (pP*_bapA_-lacZ)* | Δ*cepI* and *rpfF_Bc_*::pSHAFT double mutant, expressing *bapR* from a rhamnose-inducible and harboring a P*_bapA_-lacZ* promoter fusion | This study |
| Plasmids |  |  |
| pP*_bapA_-lacZ* | pSU11Tp containing a *bapA* promoter region fused to *lacZ* | [8] |
| pRK2013 | RK2 derivative, *mob^+^ tra*^+^ *ori* ColE1; Km^r^ | [9] |
| pSU11 | promoter probe vector; Gm^r^ | [8] |
| pSU11Tp | pSU11 derivative harboring a dhfr cassette from pRN3, Tp^r^ |  |
| pSC200 | for driving the expression of a targeted gene using the rhamnose-inducible *PrhaB* promoter. | [10] |
| pEX18Gm | *oriT^+^ sacB^+^;* pUC18 MCS, gene replacement vector; Gm^r^ | [11] |
| pGEMT-easy | cloning vector for PCR products; Amp^r^ | Promega |
| pNS-bapR | pEX18 containing an internal fragment of *bapR* for insertional mutagenesis | This study |

Antibiotic-resistance of strains or plasmids: ampicillin (Amp^r^), chloramphenicol (Cm^r^), gentamicin (Gm^r^), kanamycin (Km^r^) and trimethoprim (Tp^r^).

**References.**

1. Hanahan D (1983) Studies on transformation of *Escherichia coli* with plasmids. J Mol Biol 166: 557–580.

2. Meselson M, Yuan R (1968) DNA restriction enzyme from *E. coli*. Nature 217: 1110–1114.

3. Simon R, Priefer U, Puhler A (1983) A broad host range mobilization system for in vivo genetic engineering: transposon mutagenesis in gram negative bacteria. Bio/Technology 1: 784–791.

4. BL H, MW M, Kahn M (2004) New recombination methods for *Sinorhizobium meliloti* genetics. Appl Env Microbiol 70: 2806–2815.

5. Romling U, Wingender J, Muller H, Tummler B (1994) A major *Pseudomonas aeruginosa* clone common to patients and aquatic habitats. Appl Env Microbiol 60: 1734–1738.

6. Gotschlich A, Huber B, Geisenberger O, Tögl A, Steidle A, et al. (2001) Synthesis of multiple *N*-acylhomoserine lactones is wide-spread among the members of the *Burkholderia cepacia* complex. Syst Appl Microbiol 24: 1–14.

7. Schmid N, Deng Y, Pessi G, Aguilar C, Carlier AL, et al. (2012) The AHL- and BDSF-dependent Quorum Sensing Systems control specific and overlapping sets of genes in *Burkholderia cenocepacia* H111. PLoS One 7: e49966.

8. Inhülsen S, Aguilar C, Schmid N, Suppiger A, Riedel K, et al. (2012) Identification of functions linking quorum sensing with biofilm formation in *Burkholderia cenocepacia* H111. MicrobiologyOpen 1: 225–242.

9. Figurski DH, Helinski DR (1979) Replication of an origin-containing derivative of plasmid RK2 dependent on a plasmid function provided in trans. Proc Natl Acad Sci. 76: 1648–1652.

10. Ortega XP, Cardona ST, Brown AR, Loutet SA, Flannagan RS, et al. (2007) A putative gene cluster for aminoarabinose biosynthesis is essential for *Burkholderia cenocepacia* viability. J Bacteriol 189: 3639–3644.

11. Hoang TT, Karkhoff-Schweizer RR, Kutchma AJ, Schweizer HP (1998) A broad-host-range Flp-FRT recombination system for site-specific excision of chromosomally-located DNA sequences: application for isolation of unmarked *Pseudomonas aeruginosa* mutants. Gene 212: 77–86.
