# Supplementary material for: The IclR-Family Regulator BapR Controls Biofilm Formation in B. cenocepacia H111
Source: PLoS One. 2014 Mar 21;9(3):e92920. doi: 10.1371/journal.pone.0092920 (PMC3962473; doi:10.1371/journal.pone.0092920)
Supplement: Table S4 — Oligonucleotides used in this study. (DOCX) [file pone.0092920.s008.docx]

**Table S4**. Oligonucleotides used in this study.

| Name of oligo | Sequence |
| --- | --- |
| bapR_F | ggatccAATCGTCACCCGACGAGAT |
| bapR_R | aagcttTTCGGCCAGCTCGATCAT |
| bapR_check | TCGAGTTTCGGAATTAGAGAGA |
| pEXcheck_F | GTGCTGCAAGGCGATTAAGT |
| CA202 | TGTCGACAACTGTAACCCCTC |
| CA203 | ACGCTCGTCGTCCTCCAG |
| CA204 | TTACACGCCGATGCGCCG |
| CA215 | ctcgagCGGGAAATATGCGAAAGAAA |
| CA216 | ctcgagTTTGTAAAAGTGCCAGTG |
| CA18 | GCCTctcgagAATCTAAACGCAGGCACGAG |
| CA39 | aagcttATAGTTGGCCACGTCCTCTTT |
| CA219 | aagcttCAACAATTCGCTTGATCAG |
| CA38 | gatcctcgagCGAATAAGGTGAACCAATCCAG |
| CA228 | aagcttCGCCCTCGCCATATTAGTTA |
| CA229 | aagcttAAAGGAAAAATGGAGCCG |
| CA230 | aagcttCAAACGTTCAACCAAACAG |
| CA231 | aagcttGAAACCATTGCCCGACTC |
| CA232 | aagcttATCGACGTGATTGCTCAA |
| CA233 | gatcaagcttCTCGTGCCTGCGTTTAGATT |
| CA234 | gatcaagcttCGCGGTGCACCATCACTC |

Restriction sites in the oligonucleotides are shown underlined.
